# S-1 plus oxaliplatin versus capecitabine plus oxaliplatin for the first-line treatment of patients with metastatic colorectal cancer: updated results from a phase 3 trial

**DOI:** 10.1186/1471-2407-14-883

**Published:** 2014-11-26

**Authors:** Seung Tae Kim, Yong Sang Hong, Ho Yeong Lim, Jeeyun Lee, Tae Won Kim, Kyu-Pyo Kim, Sun Young Kim, Ji Yeon Baek, Jee Hyun Kim, Keun-Wook Lee, Ik-Joo Chung, Sang-Hee Cho, Kyung Hee Lee, Sang Joon Shin, Hye Jin Kang, Dong Bok Shin, Jae Won Lee, Sook Jung Jo, Young Suk Park

**Affiliations:** Department of Medicine, Samsung Medical Center, Sungkyunkwan University School of Medicine, 81 Irwon-ro, Gangnam-gu, Seoul, 135-710 Korea; Department of Oncology, Asan Medical Center, University of Ulsan College of Medicine, Seoul, Korea; Centre for Colorectal Cancer, National Cancer Center, Goyang, Korea; Department of Internal Medicine, Seoul National University Bundang Hospital, Seoul National University College of Medicine, Seongnam, Korea; Department of Internal Medicine, Chonnam National University Hwasun Hospital, Chonnam National University Medical School, Jeollanamdo, Korea; Department of Internal Medicine, Yeungnam University Hospital, Yeungnam University College of Medicine, Daegu, Korea; Department of Internal Medicine, Cancer Metastasis Research Centre, Yonsei University College of Medicine, Seoul, Korea; Department of Internal Medicine, Korea Cancer Centre Hospital, Korea Institute of Radiological and Medical Sciences, Seoul, Korea; Department of Internal Medicine, Gachon University Gil Hospital, Incheon, Korea; Department of Statistics, Korea University, Seoul, Korea; Biostatistics, ICON Clinical Research, Seoul, Korea

**Keywords:** Capecitabine, S-1, Colorectal cancers

## Abstract

**Background:**

We report updated progression-free survival (PFS) and overall survival (OS) data from a trial that compared capecitabine plus oxaliplatin (CapeOX) versus S-1 plus oxaliplatin (SOX) for the first-line treatment of metastatic colorectal cancer.

**Methods:**

This trial was a randomized, two-armed, non-inferiority phase 3 comparison of CapeOX (capecitabine 1000 mg/m2 twice daily on days 1–14 and oxaliplatin 130 mg/m2 on day 1) versus SOX (S-1 40 mg/m2 twice daily on days 1–14 and oxaliplatin 130 mg/m2 on day 1). The primary end point was to show non-inferiority of SOX relative to CapeOX in terms of PFS. Thus, a follow-up exploratory analysis of PFS and OS was performed.

**Results:**

The intention to treat (ITT) population was comprised of 340 patients (SOX arm: 168 and CapeOX arm: 172). The updated median PFS was 7.1 months (95% CI 6.4-8.0) in the SOX group and 6.3 months (95% CI 4.9-6.7) in the CapeOX group (hazard ratio [HR], 0.83 [0.66-1.04], p = .10). The median OS was 19.0 months (95% CI 15.3-23.0) in the SOX group and 18.4 months (95% CI 14.1-20.7) in the CapeOX group (HR, 0.86 [0.68-1.08], p = .19). Subgroup analyses according to principal demographic factors such as sex, age, ECOG (Eastern Cooperative Oncology Group) performance status, primary tumor location, measurability, previous adjuvant therapy, number of metastatic organs, and liver metastases showed no interaction between any of these characteristics and the treatment.

**Conclusions:**

Updated survival analysis shows that SOX is similar to CapeOX, confirming the initial PFS analysis. Therefore, the SOX regimen could be an alternative first-line doublet chemotherapy strategy for patients with metastatic colorectal cancer.

**Trial registration:**

NCT00677443 and May 12 2008

## Background

Fluoropyrimidines (5FU) have remained the most commonly prescribed agents for gastrointestinal cancer, including colorectal cancer (CRC). 5FU is administered as a continuous infusion by a portable pump or by an inserted chemo-port, methods that provide continuous exposure and modest improvement in efficacy. However, continuous infusion is inconvenient and unsafe [1, 2]. Thus, the development of oral FU (capecitabine and S-1) has opened new possibilities for the treatment of gastrointestinal tumors, especially gastric cancers [3–5]. For the first-line treatment of patients with metastatic CRC, oxaliplatin plus either fluorouracil or capecitabine has been one of the reference doublet cytotoxic chemotherapy strategies [6, 7]. S-1 is a novel oral FU consisting of a 5FU prodrug, tegafur, the dihydropyrimidine dehydrogenase inhibitor, 5-chloro-2, 4-dihydroxypyrimidine, and the orotate phosphoribosyl transferase inhibitor, potassium oxonate, which suppresses the gastrointestinal toxicity of tegafur [8]. Although several trials have shown the feasibility and efficacy of S-1 plus oxaliplatin (SOX) as an upfront chemotherapy for metastatic CRC [9, 10], S-1 and capecitabine have not been directly compared when either is combined with oxaliplatin. To address this dearth of information, we conducted our initial randomized, non-inferiority phase III trial of SOX versus capecitabine plus oxaliplatin (CapeOX) for the first-line treatment of patients with metastatic colorectal cancer [11]. We found the S-1 group to have nearly 2 months longer PFS than the capecitabine group, suggesting that the SOX regimen could be an alternative first-line doublet chemotherapy strategy for patients with metastatic CRC. However, which particular S-1s can be used as substitutes for capecitabine may be controversial in CRC. Thus, we intend to update the overall survival (OS) and progression free survival (PFS) results, and we intend to conduct exploratory analyses to determine whether the effect of S-1 on these end points appears to vary in selected patient groups.

## Methods

### Study design

This was a randomized, open-label, multicenter phase 3 study [11]. The institutional review boards of all participating institutions approved the study protocol. Written, informed consent was required for participation.

To be eligible, patients with metastatic colorectal cancer were required to have histologically confirmed adenocarcinoma, measurable or assessable lesions, an Eastern Cooperative Oncology Group (ECOG) performance status (PS) of 0–2, no previous chemotherapy or immunotherapy in a metastatic setting, adequate hematological, hepatic, and renal function, and be 18 years of age or older. Adjuvant chemotherapy or radiotherapy was permitted if it had been completed at least 6 months before enrollment. We randomly assigned eligible patients to either CapeOX or SOX in a one-to-one ratio. Randomisation was done centrally with a computer-generated sequence and a permutation block technique that ensured equal distribution of patients on the basis of primary tumor site (colon vs. rectum), history of previous adjuvant or neoadjuvant treatment, and the presence of measurable lesions.

### Procedures

All treatment cycles were administered every three weeks. We administered oral S-1 (40 mg/m2) twice a day on days 1–14, oral capecitabine (1000 mg/m2) twice a day on days 1–14, and oxaliplatin (130 mg/m2) on day 1 as a 2-h intravenous infusion. As many as nine cycles of oxaliplatin-containing chemotherapy were provided, except in instances of disease progression, unacceptable toxicity, or patient refusal. Maintenance chemotherapy with S-1 or capecitabine was permitted after discontinuation of oxaliplatin. Treatment responses were assessed every three cycles (9 weeks) during study treatments or sooner if needed for documentation of disease progression. Objective tumor responses were independently reviewed according to the Response Evaluation Criteria In Solid Tumors (RECIST; version 1.0).

### Statistical analyses

For the primary efficacy analysis, in which we aimed to assess the non-inferiority of SOX to CapeOX in terms of PFS (time to progression or death), we assessed all patients allocated to the treatment group (intention to treat population), and we also did a per-protocol analysis in those who received protocol treatments without major violations. PFS at 15 months in both groups was assumed to be 38%, and the low non-inferiority limit was set as −13%, corresponding to a HR of 1.43. On the basis of these conditions, 192 events were needed for a one-sided type I error of 5% and a power of 80%. Assuming a 10% loss, we needed 344 patients. Previous reports were made at the August 31, 2011 cut-off date for data collection. These updated data were collected at the cut-off date of December 24, 2013. The survival data were assessed with the Kaplan-Meier method. We estimated the hazard ratio (HR) and corresponding 95% CI using the Cox proportional hazard regression model. Also, we analyzed post-progression survival (PPS; the duration of survival after disease progression to study medication) among two groups.

## Results

### Patients

**Figure 1:**
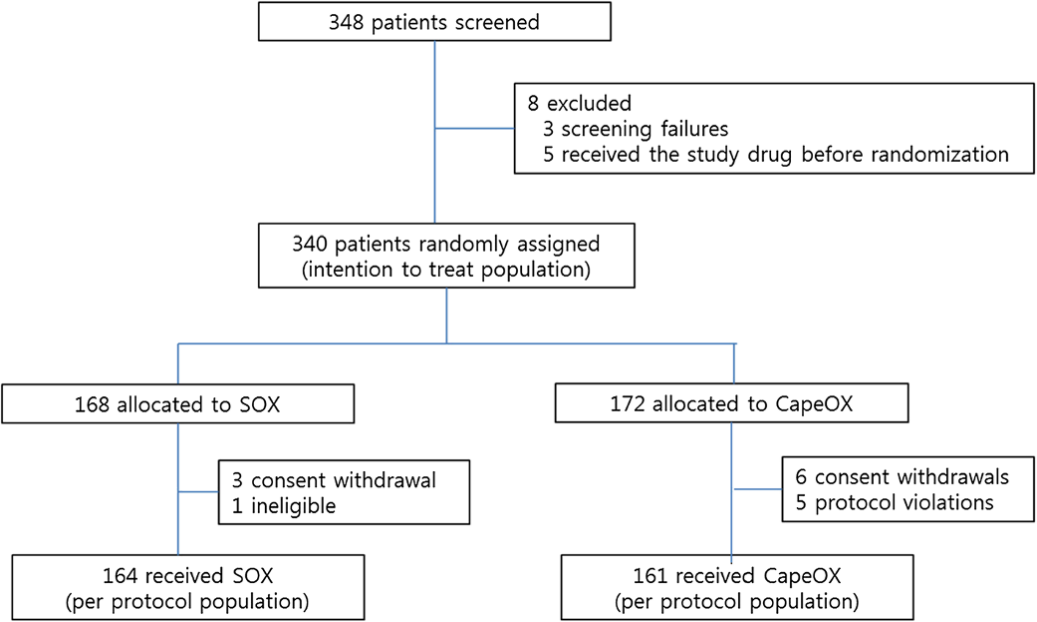
**CONSORT diagram.**

Between May 14, 2008 and September 23, 2009, we enrolled 348 patients from 11 institutions. We randomly assigned 340 patients who met the eligibility criteria to treatment (ITT). Of the 340 patients, 168 were assigned to the SOX group, and 172 were assigned to the CapeOX group (Figure 1). This updated analysis used data collected by December 24, 2013, by which time, 301 (SOX group: 150, CapeOX group: 151) PFS events, and 279 (134; SOX group, 145; CapeOX group) OS events had occurred. In the previous analysis, 207 (98; SOX group, 109; CapeOX group) PFS events, and 179 (84; SOX group, 95; CapeOX group) OS events had occurred. Of note, the baseline characteristics were much the same between the two groups (Table 1).

**Table 1:** **Baseline characteristics (intention to treat population)**

	SOX (***n*** = 168)	CapeOX (***n*** = 172)
Primary site		
Colon	109 (65%)	108 (63%)
Rectum	59 (35%)	64 (37%)
Sex		
Male	109 (65%)	102 (59%)
Female	59 (35%)	70 (41%)
Age		
≤65 years	121 (72%)	126 (73%)
>65 years	47 (28%)	46 (27%)
ECOG performance status		
0-1	164 (98%)	168 (98%)
2	4 (2%)	4 (2%)
Previous (neo)adjuvant therapy		
Yes	37 (22%)	38 (22%)
No	131 (78%)	134 (78%)
Site of metastasis		
Liver metastasis	105 (63%)	111 (65%)
Non-liver metastasis	63 (37%)	61 (35%)
Number of metastatic organs		
One organ	65 (39%)	49 (29%)
Two organs	61 (36%)	70 (41%)
Three or more organs	42 (25%)	53 (31%)
Measurability		
Measurable lesions	155 (92%)	155 (90%)
Assessable lesions only	13 (8%)	17 (10%)

### Progression-free survival (PFS) and post-progression survival (PPS)

**Figure 2:**
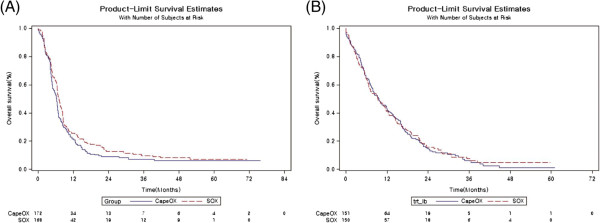
**Kaplan-Meier curves of progression-free survival (PFS) (A) and post-progression- survival (PPS) (B).**

In the SOX group with media 17.91 follow-up, the median PFS was 7.1 months (6.4-8.1), and the corresponding value in the CapeOX group with median 16.41 follow-up was 6.3 months (4.9-6.7, intention to treat [ITT] population) (Table 2 and Figure 2). The HR comparing PFS between the two groups was 0.83 (95% CI 0.66-1.04, *p* = .10). For the ITT population, the median post-progression survival (PPS) was 9.3 months in the SOX group compared to 9.5 months in the CapeOX group, with a corresponding HR of 0.97 (95% CI 0.76-1.23, *p* = .81) (Table 2 and Figure 2).

**Table 2:** **Survival outcomes**

	Intention to treat population			Per-protocol population		
	SOX	CapeOX	Effect size (95% CI); ***p*** value	SOX	CapeOX	Effect size (95% CI), ***p*** value
Number of patients	168	172		164	161	
Median PFS	7.1 (6.4-8.1)	6.3 (4.9-6.7)	0.83 (0.66-1.04), *p* = .10	6.9 (6.4-7.9)	6.3 (5.2-6.7)	0.84 (0.67-1.06), *p* = .13
Median OS	19.0 (15.3-23.0)	18.5 (14.1-20.8)	0.86 (0.68-1.08), *p* = .19	19.1 (15.0-23.0)	17.6 (14.1-20.5)	0.85 (0.67-1.08), *p* = .17
Number of patients	150	151		147	145	
Median PPS	9.3 (6.7-11.6)	9.5 (7.4-12.1)	0.97 (0.76-1.23), *p* = .81	9.3 (6.9-11.6)	9.6 (7.4-12.1)	0.96 (0.75-1.23), *p* = .75

Data are represented as n (%), time in months (95% CI) or effect size (95% CI). SOX, S-1 plus oxaliplatin; CapeOX, Capecitabine plus oxaliplatin; PFS, progression-free survival; PPS, post-progression survival, OS = overall survival.

### Overall survival (OS)

**Figure 3:**
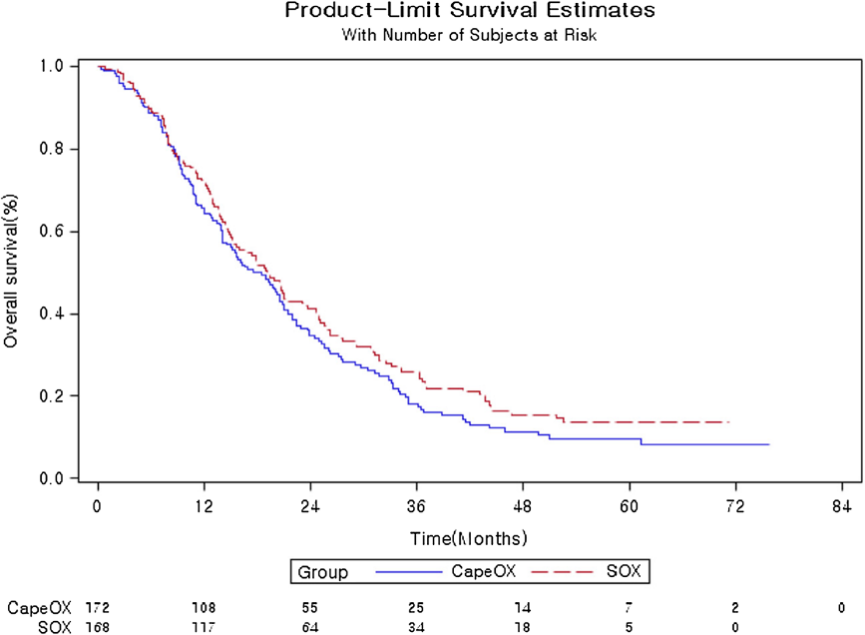
**Kaplan-Meier curves of overall survival (OS).**

The OS data in the ITT population as of December 24, 2013 are shown in Table 2. The corresponding Kaplan-Meier curves for OS are shown in Figure 3.

For the ITT population, median OS was 19.0 months in the SOX group compared to 18.5 months in the CapeOX group, with a corresponding HR of 0.86 (95% CI 0.68-1.08).

### Subgroup analysis

**Figure 4:**
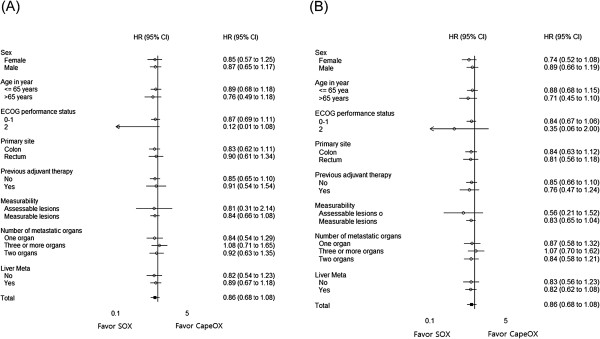
**Subgroup analysis of overall survival (A) and progression-free survival (B) for the intention to treat population.**

OS and PFS in assigned patients were analyzed according to sex, age, ECOG PS, primary tumor site, previous adjuvant therapy, measurability, number of metastatic organs, and liver metastasis. There was no interaction between the treatment and any of these factors (Figure 4).

## Discussion

The primary analysis of this study showed that SOX is non-inferior to CapeOX in terms of PFS, OS, and response rate as a first-line chemotherapy in patients with metastatic colorectal cancer (CRC) [11]. To the best of our knowledge, this study is the first large, phase III clinical trial that directly compares SOX with CapeOX in metastatic CRC. This updated analysis also demonstrates that SOX and CapeOX have statistically similar PFS and OS, confirming the conclusion reached in our previous publication.

In this updated analysis, patients in the CapeOX group had a PFS of 6.3 months and an OS of 18.5 months, which are similar values to those reported in previous trials [6, 12–14]. Likewise, the SOX group in our analysis showed favorable results, which are similar to values recorded for CapeOX or FU plus oxaliplatin plus Leucovorin (FOLFOX) treatment in previous studies [12–14]. Based on our updated analysis with a long-term follow-up, no significant differences were observed in PFS and OS between the SOX and CapeOX groups, which is consistent with previous gastrointestinal cancer studies [15–18]. In addition, a meta-analysis by Zhang et al. reported that both the S-1 and capecitabine-based regimens were equally active and well tolerated in patients with gastric cancer and CRC [19].

In this updated analysis, we also investigated the post-progression survival (PPS) for both treatment groups. In cancer treatment, the goal is to prolong survival and/or to improve the patient’s quality of life. In the era of multiple lines of chemotherapy, therapeutic options have been expanded, especially in CRC. In breast cancer, lung cancer, and CRC, OS becomes more associated with PPS than PFS [20–23]. In CRC, the association of PPS with OS may be due to the increasing number of active compounds available, or to the available second or third-line therapies. In this analysis, there was no significant difference in PPS between the SOX and CapeOX groups (Table 2). These findings suggest that effective treatments, equal in quality, were provided to both groups after the failure of the first-line therapy. The dose of S-1 in this study was 80 mg/m2 per day, which is higher than doses used in the reference trial that combined S-1 with oxaliplatin. As mentioned in a previously published report, this dose finding explains the high frequency of adverse events in SOX-treated patients. However, the high frequency of adverse events in the SOX group might not affect subsequent treatment strategies.

In patients with metastatic CRC, the addition of bevacizumab to the first-line chemotherapy has improved survival. Previous studies have shown that bevacizumab plus FOLFOX, FOLFIRI (leucovorin, fluorouracil, and irinotecan), or CapeOX improves PFS [12, 14, 24–26]. However, the role of bevacizumab with SOX has not been established. Recently, Yamada et al. showed that bevacizumab plus SOX was non-inferior to bevacizumab plus FOLFOX as a first-line treatment for metastatic CRC with respect to PFS [27]. Thus, bevacizumab plus SOX might be a possible option as a standard first-line treatment, although S-1 and capecitabine have not been directly compared when either is combined with bevacizumab and oxaliplatin.

Our updated analysis confirms that a combination of S-1 and oxaliplatin can be considered as an alternative doublet chemotherapy strategy to CapeOX. Further investigation is needed to explore its potential when used together with other targeted agents or as adjuvant chemotherapy.

## Conclusion

Updated survival analysis shows that SOX is similar to CapeOX, confirming the initial PFS analysis. Therefore, the SOX regimen could be an alternative first-line doublet chemotherapy strategy for patients with metastatic colorectal cancer.

### Ethics statement

The Ethics Committee at Samsung Medical Center approved the study in accordance with the Declaration of Helsinki. All individuals gave written informed consent for participation in the study.
